# Unconscious Other’s Impression Changer: A Method to Manipulate Cognitive Biases That Subtly Change Others’ Impressions Positively/Negatively by Making AI Bias in Emotion Estimation AI

**DOI:** 10.3390/s22249961

**Published:** 2022-12-17

**Authors:** Kyosuke Futami, Sadahiro Yanase, Kazuya Murao, Tsutomu Terada

**Affiliations:** 1 Graduate School of Information Science and Engineering, Ritsumeikan University, 1-1-1 Nojihigashi, Kusatsu 525-8577, Shiga, Japan; 2 Digital Spirits Teck, Kusatsu 525-8577, Shiga, Japan; 3 Graduate School of Engineering, Kobe University, Kobe 657-8501, Hyogo, Japan

**Keywords:** bias in AI, emotion estimation AI, information presentation, cognitive biases, psychological effect, impression of people, human–computer interaction

## Abstract

Artificial Intelligence (AI) for human emotion estimation, such as facial emotion estimation, has been actively studied. On the other hand, there has been little research on unconscious phenomena in cognition and psychology (i.e., cognitive biases) caused by viewing AI emotion estimation information. Therefore, this study verifies RQ “Do people have a cognitive bias in which impressions of others (i.e., how to see and feel about others) are changed by viewing biased AI’s emotion estimation information? If it exists, can impression manipulation methods that intentionally use this cognitive bias be realized?” The proposed method for verification makes the emotion estimation system biased so as to estimate emotion more positively/negatively than AI without bias. A prototype system was implemented. Evaluation using video showed that the presentation of biased emotion estimation information causes a phenomenon that quickly and unconsciously changes the way people see and feel others’ impressions, which supported the RQ. Specifically, viewing information that estimated others’ emotions more positively/negatively caused the phenomenon in which the user’s self-judgment was overridden and others’ impressions of emotions, words, and actions were perceived more positively/negatively. The existence of this phenomenon and method indicates that biased emotion estimation AI has the potential to both cause adverse effects on people and support people for good purposes through the manipulation of their impressions. This study provides helpful insights for the design and use of emotion estimation AI considering cognitive biases.

## 1. Introduction

In recent years, systems using artificial intelligence (AI) that estimate a person’s inner state are becoming increasingly popular in the real world. Among these, technology for human emotion estimation has been actively studied. Such examples include the technology using faces in camera images [1], the technology using speech audio signals [2,3], the technology using text [4], and the technology using brain activity based on EEG signals [5]. These emotion estimation technologies can be installed in information devices (e.g., wearable glasses, mobile devices) and can be used in many situations where people see each other on either information devices or in person. Since it is often helpful to estimate the emotional state of others and oneself, it can be assumed that emotion estimation technology will be used in society to assist people in making decisions.

On the other hand, little research has investigated whether or not people’s cognitive decisions are influenced by viewing AI’s emotion estimation information and whether or not such influences can be intentionally manipulated. This investigation is considered important in light of the following two contexts. (1) First, because computer information presentation can unintentionally affect human cognition and psychology due to unconscious phenomena (e.g., cognitive biases, psychological effects, and illusions), many recent studies have shown the importance of understanding the existence and manipulation methods of such unconscious phenomena [6,7,8,9,10,11]. For example, a previous study of information presentation systems for wearable biosensors discovered a phenomenon in which the user’s mind and body unconsciously changes in accordance with the presented sensor value, even if the sensor value is different from the actual value due to a bias in the sensor value presentation algorithm (e.g., changes in anxiety with heart rate sensor presentation [12,13,14], changes in physical load with myoelectric sensor presentation [15]). The previous study also discovered a method that presents modified sensor value (i.e., false value ) to manipulate this phenomenon, which indicated that this phenomenon and method could be used for good to support the user and could be misused to harm the user. (2) Next, in recent years, there have also been examples of unintended negative effects on people and society caused by biased output from AI systems [16,17,18,19,20]. Such examples include a hiring assistance AI that estimates things like male privilege (i.e., sexism) [21,22], a criminal behavior estimation AI that makes adverse predictions for black prisoners [20,22,23], image labeling AI that estimates black men as gorillas [19,23], and AI chatbots that perform racist speech and behavior [18]. Since there is a high probability that an emotion-estimation AI that makes biased estimations will accidentally (or intentionally) spread in society as in the AI systems that have already caused problems, it can be assumed that people’s anticipation in advance of the unconscious phenomena that emotion-estimation AI will cause on people will help prevent the spread of unanticipated damage. Indeed, concerns about the adverse effects of emotion-estimation AI are often the topic of popular scientific articles by experts (e.g., Time to regulate AI that interprets human emotions [24]). From these two backgrounds, it is important to investigate and clarify the existence of cognitive bias caused by viewing AI’s emotion estimation information and the manipulation methods of such cognitive bias.

The research questions for this study are as follows. “RQ. Do people have a cognitive bias in which impressions of others (i.e., how to see and feel about others) are changed by viewing biased AI’s emotion estimation information? If it exists, can impression manipulation methods that intentionally use this cognitive bias be realized?”.

**Figure 1:**
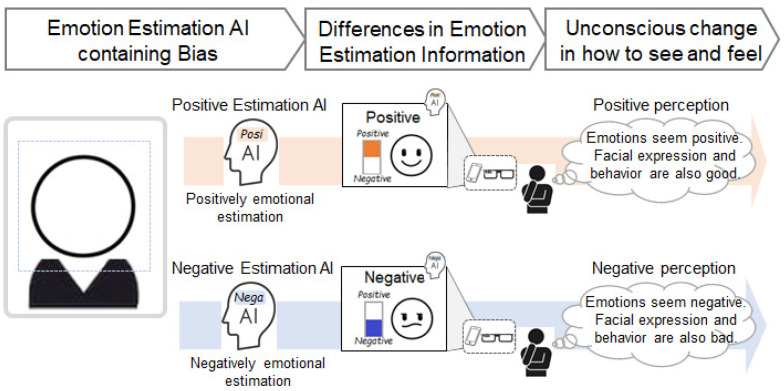
Phenomena assumed in this study. When a person views another person, viewing the more negatively (or more positively) estimated emotion estimation results will cause a phenomenon in which the person perceives that the other person’s emotion is more negative (or more positive).

**Figure 2:**
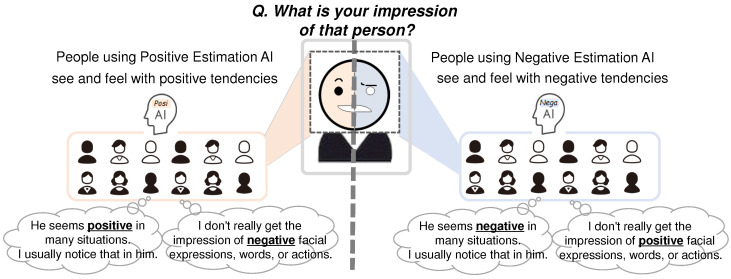
One of future forecast images. People are affected according to the bias of the emotion estimation AI.

**Figure 3:**
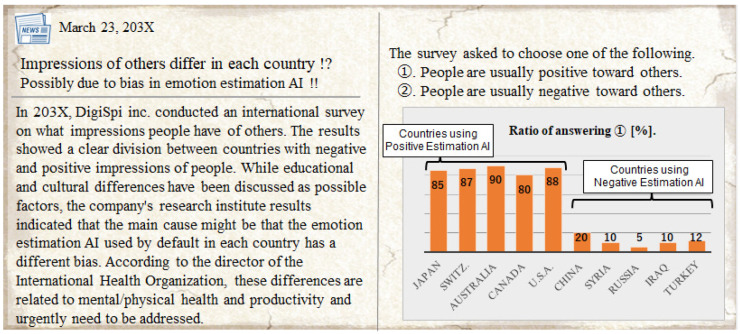
One of future forecast images. An example of a newspaper report in which the cause of differences in the way people see and feel others from country to country is the diffusion of emotion estimation AI with different biases from country to country.

Based on previous examples in which human cognition and psychology are influenced by viewing biometric sensor information such as heartbeats and myoelectric values, it is assumed that human cognition and psychology also change unconsciously, consistent with the presented AI’s emotion estimation information. The phenomena assumed in this study are shown in Figure 1. For example, when a person views another person, viewing the more negatively (or more positively) estimated emotion estimation results will cause a phenomenon in which the person perceives that the other person’s emotion is more negative (or more positive). Since different emotion estimation AI from different providers have different biases, people are assumed to be affected according to the bias of the emotion estimation AI. In addition, emotional impressions may have spillover effects. For example, suppose people have a more negative (or more positive) perception of another person’s emotions due to AI-estimated information. In that case, people may also have a more negative (or more positive) perception of another person’s words, actions, and facial expressions. If such cognitive biases exist, the manipulation of impressions of a particular person may occur, either unintentionally (i.e., accidentally) or intentionally. Such examples of particular persons include celebrities, politicians, or communication partners online or in person. Such a phenomenon may lead to negative issues for people and society, such as political inducements, purchase inducements, mental ill-health, disagreements, and interpersonal conflicts. On the other hand, this phenomenon may be used for good purposes. For example, if there is a technology that allows people to unconsciously change the way they see and feel others, it will have a positive effect on activities that involve or cooperate with others. Figure 2 and Figure 3 show one of the future forecast images.

Therefore, the purpose of this study was to verify the RQ mentioned above. Specifically, we aim to show whether impressions of others can be influenced or not by an emotion estimation AI that has a bias in the estimation results and to show whether a method to intentionally manipulate this influence can be implemented or not. For the verification, we propose a method called “Mindless impression changer”, which presents emotion estimation information to unconsciously change the impressions of others. We define an AI that estimates emotions as per the prepared training dataset as a standard estimation AI. The proposed method replicates the emotion estimation system that has bias and estimates emotion more positively (or negatively) compared to the normal estimation AI. The proposed method aims to induce the phenomenon of perceiving impressions of others in a more positive direction or more negative direction. We implemented a prototype system and verified our research question. The results supported the hypothesis of this study. The results showed a phenomenon in which the way people see and feel others’ impressions changes to be consistent with the presented emotion estimation information even if there is a bias in the emotion estimation information. The results also showed the feasibility of the method that easily and unconsciously changes the way people see and feel others’ impressions by using this phenomenon. The results of our study provided important considerations for the design and the use of emotion estimation AI.

## 2. Related Work

The following studies have been conducted to clarify the existence and manipulation method of unconscious phenomena (e.g., cognitive biases, psychological effects, and illusions) that computer information presentation causes in people.

Some studies focused on cognitive biases related to how people are seen. For changing the user’s emotional state, Yoshida et al. [25] proposed a feedback method that modifies the user’s facial expression to be more positive than the actual one. For improving the quality of collaborative work, Suzuki et al. [26] propose a method that changes the facial expressions of dialogue partners from actual ones. On the other hand, our study focuses on the cognitive bias caused by changing only the emotion estimation information without changing the person’s appearance.

Some methods focused on cognitive biases related to changing the state and ability of the mind and body. Costa et al. [12,13,14] discovered that the user’s physical and mental state (e.g., anxiety level, cognitive performance) unconsciously changed so as to match the presented heart rate sensor information, even though the presented heart rate is different from the actual heart rate. They also demonstrate a method of intentionally manipulating this phenomenon. In addition, for improving the user’s ability to use mind and body in nervous situations in sports, there is a method that presents success-conditioned sounds (Futami et al. [27]) and a method that presents a pseudo-success experience in VR space (Tagami et al. [28]). Some studies have focused on other cognitive biases. Duarte et al. have revealed a psychological phenomenon like the placebo effect in which obtaining power-up items during a game actually changes the user’s cognitive performance as well [29]. There is a method for lowering psychological stress during discussions by modifying the voices of oneself and others [9] and a visual interface to improve the recall speed of memorization learning [30].

Several studies focused on cognitive biases related to concentration. To improve concentration and productivity at work, there are a method that presents a work productivity log based on framing effects (Kim et al. [31]), a method that presents modified time elapsed speed on a digital clock (Ban et al. [32]), and a method that detects the decrease in user’s concentration with machine learning and modulating the sound of the video teaching material (Arakawa et al. [33]). Several methods focus on sensory-related cognitive biases. To manipulate the elapsed time sensation, some methods use tactile stimuli using wrist-worn devices [10,34], auditory stimuli of information devices [35], and visual icons on a head-mounted display [11,36]. To manipulate load perception such as fatigue and weight of things, there is a method that presents myoelectricity sensor information that differs from the actual measured sensor value [15,37] and a method that changes the appearance color of the object to be lifted [38]. To manipulate the user’s feeling of fullness, there is a method that changes the visual size of food to eat with VR (Narumi et al. [39]) and a method that changes the apparent size of the plate (Adams et al. [6]).

Several methods focus on cognitive biases related to behavior and choice. To increase motivation for daily walking exercise, Futami et al. [8] proposed a method that creates a virtual competitor in a step-logging competition and manipulates the psychological phenomenon caused by competition. Takeuchi et al. [40] showed that a user’s meal selection and taste preference are affected by other people’s evaluation (e.g., likes, good) of the user’s meal content that is posted in a system similar to SNS, and they also proposed a method that induces users to select healthy meals by presenting other people’s modified evaluations. For controlling tourists flow and reducing congestion, Shen et al. [41,42] proposed a method that changes the tourist route customization screen and store rankings. For inducing the behavior of not missing the train, Futami et al. [43] proposed a method that shows the train schedule that is different from the actual one. Some studies indicated that visual information on information presentation screens such as HMDs and smartphones could affect what object the user pays attention to in real life (Isoyama et al. [44,45]). Interactive mannequins [46] and ambient light systems [47] also affect what object the user pays attention to. Some studies make online behavior thoughtful. For improving users’ thoughtfulness in posting a comment online, Menon et al. [48] proposed a method that partitions the text field to write a comment and a method that displays the number of comment words. For inhibiting users’ thoughts and behavior of spreading fake news on social media, Yaqub et al. [49] proposed a method that presents trust indicators along with the news.

In recent years, there have also been examples of unintended negative effects on people and society caused by biased output from AI systems. For example, an Amazon-developed hiring assistance AI made estimates with a sexist bias (e.g., male-preferential, female-discriminatory) [21,22], a Google Photos image labeling AI classified dark-skinned people as gorillas [19,23], a criminal behavior estimation AI made a negative estimation for black prisoners [20,22,23], the face unlock feature of Apple’s iPhone X could not distinguish between two different Asians [23,50], an automatic soap dispenser could not recognize black skin hands [23,51], and Microsoft’s AI chatbot presented racist thoughts, words, and actions [18]. Some AIs that could cause problems in the future have been reported, e.g., an AI that estimates an individual’s sexual orientation based on facial features [23,52]. AI systems often have harmful biases against minorities. For these biases, there are investigations into the causes of bias in a real-world application [16], bias diagnostic methods [53], and mitigation and management methods of bias [17]. Some cognitive biases caused by the perception of AI estimation results are similar to the anchoring effect. In the anchoring effect, a phenomenon occurs in which a person’s cognitive judgments criteria become closer to the pre-presented information. For example, when judging the price of an automobile, a person’s judgment changes (i.e., the judgment becomes closer to the presented information) between the two conditions of viewing a higher price or a lower price. This is because people adjust their judgments using the preceding information as a starting point and unconsciously recall and collect information that reinforces the preceding information. This phenomenon has been shown to be robust and difficult to resolve because this phenomenon occurs regardless of the following factors: expertise in the task (e.g., realtor’s knowledge of real estate values) [54], prior warning (e.g., explanation of psychological phenomena) [55], and reward (e.g., reward offered based on the degree of accuracy of judgment) [56]. Even in the case of cooperative work with AI, there are examples of this phenomenon similar to the anchoring effect in which human judgments are affected by viewing the AI’s estimated results, and the importance of understanding and controlling this phenomenon is shown [57,58]. Our study focuses on the cognitive bias caused by emotion estimation AI based on these previous studies.

The existence and manipulation methods of the phenomena revealed by these previous studies have provided important implications for using and designing information devices in many fields such as human–computer interaction, wearable computers, and UBICOMP. Revealing what happens to users due to the use of information devices is important for spreading information devices. The necessity of the discovery of the existence and mitigation methods of cognitive biases that are caused by the use of information systems is emphasized [7]. Our study is expected to provide useful insights into the design and use of information devices.

## 3. Method

In this section, we describe the hypothesis of this study and the methods to manipulate the cognitive biases caused by the emotion estimation AI.

### 3.1. Hypothesis

As described in Section 1, the research questions for this study are as follows. “RQ. Do people have a cognitive bias in which impressions of others (i.e., how to see and feel about others) are changed by viewing biased AI’s emotion estimation information? If it exists, can impression manipulation methods that intentionally use this cognitive bias be realized? For example, impressions of a particular person can be manipulated in a more negative (or positive) direction by using AI emotion estimation information”.

The following cases are assumed.

Case 1. This phenomenon does not exist. In other words, knowing AI emotion estimation information does not change or affect people’s cognition and psychology. In this case, we can report that there is no concern that this phenomenon will cause harmful problems.

Case 2. This phenomenon exists, but it occurs with an unpredictable trend. In this case, this phenomenon cannot be intentionally manipulated. For example, if a person’s cognitive and psychological changes occur randomly when presented with a particular pattern of AI emotion estimation information, this phenomenon cannot be manipulated.

Case 3. This phenomenon exists and occurs with a predictable tendency. In this case, this phenomenon can be intentionally used or suppressed. For example, if a people’s cognitive/psychological change occurs with a predictable trend when presented with a specific pattern of AI emotion estimation information, this phenomenon can be intentionally manipulated.

### 3.2. Impression Manipulation Method Using AI Emotion Estimation Systems

In this paper, we use a system that estimates the subject’s emotions on a positive or negative scale to test whether it is possible to manipulate the impressions in a more positive or more negative direction.

We designed the following two types of emotion estimation AIs.

#### 3.2.1. Positive Estimation AI

This AI tends to estimate emotions in a more positive direction. We define the AI that estimates as per the prepared training dataset as the normal estimation AI. Compared to the normal estimation AI, the positive estimation AI estimates emotions in a more positive direction. For example, suppose the average of a particular facial expression X in the training dataset made by people is 50 points on a scale of 0 to 100 (0: very negative, 100: very positive). In that case, the normal estimation AI will estimate that facial expression X as 50 points. In contrast, the positive estimation AI will estimate more positively (e.g., 90 points) for that facial expression X. The detailed values are adjusted for each experiment. We assume that this AI causes the phenomenon of making users perceive others’ emotions more positively.

#### 3.2.2. Negative Estimation AI

This AI tends to perform emotion estimation more negatively. Compared to the normal estimation AI, the negative estimation AI estimates emotions in a more negative direction. For example, while the normal estimation AI estimates the degree of positivity of a particular facial expression X as 50 points on a scale 0 to 100, the negative estimation AI produces a more negative estimation result (e.g., 25 points) for that facial expression X. The detailed values are adjusted for each experiment. We assume that this AI causes the phenomenon of making users perceive others’ emotions more negatively.

#### 3.2.3. Implementation

**Figure 4:**
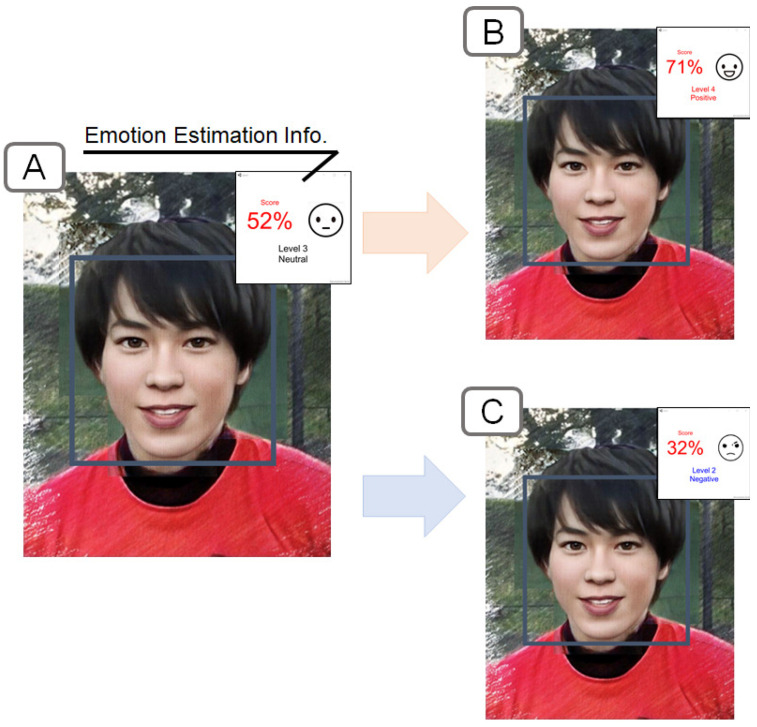
(**A**) Example of emotion estimation information, (**B**) example of the positive estimation information presentation, and (**C**) example of the negative estimation information presentation.

A prototype system of the proposed method was implemented. The presentation screen of the emotion estimation information is shown in Figure 4. The emotion estimation information about the target person in the window is displayed in the emotion estimation information window. The emotion estimation information window can be placed at any position and show emotion scores on a scale from 0 to 100 points for a specific facial expression. The text and image icons change according to the score.

In order to estimate the emotion of the person in the media content in our evaluation experiments, this system was designed to calculate the emotion estimation information in the loaded video file. The entire prototype system consists of an emotion estimation application and a laptop PC. The emotion estimation function was implemented in Python, and the information presentation screen was implemented in Unity. Facial-Expression-Keras was used to calculate the subject’s emotion estimation information in the video files. The extended Cohn–Kanade dataset (CK+) [59] and Coding facial expressions with Gabor wavelets (JAFFR) [60] were used when creating the emotion estimation model. As for indicators for emotion estimation, happiness related to the positive scale and anger related to the negative scale can be selected, and each indicator is estimated on a scale of 0 to 100. This system selects an appropriate indicator of emotion estimation for each experiment and adjusts to estimate with biased tendencies by setting the minimum and maximum estimates for each experiment.

## 4. Evaluation 1: Verification of Impression Manipulation of a Person with Negative Facial Expressions

Evaluation 1 verified the hypotheses on a person with negative facial expressions and behaviors. The 44 subjects were Japanese. Subjects were college students in their early 20s and were collected through an open recruitment process at the university.

### 4.1. Negative Facial Expression Person

**Figure 5:**
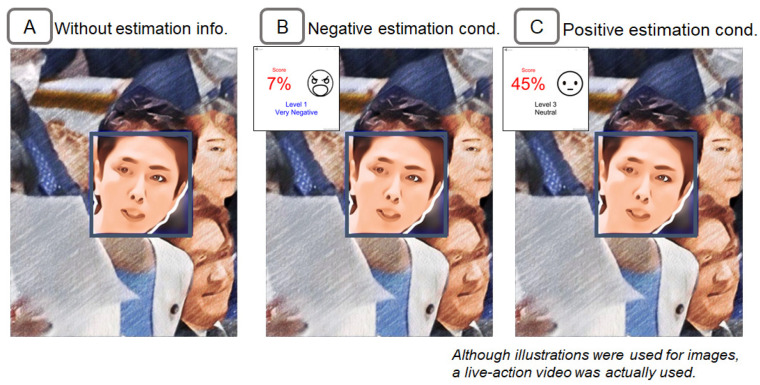
Presentation information for the negative facial expression politician in Experiment 1. (**A**) Screen with no emotion estimation information, (**B**) screen with negative estimation information, and (**C**) screen with positive estimation information. Although this figure is an illustration, live-action video was used.

The subject of the impression manipulation was a politician who was critical of the other party in a debate situation. The debate situation was selected from a TV broadcast of the Japanese government’s Diet. The entire video was about 30 s long. In the video, one politician was shown in the center of the picture, as shown in Figure 5A. The politician’s facial expression was negative. She blames her opponent for her words and actions (e.g., waves her arms). For example, the words include the following.

“You made a mistake in the voting behavior of the members of the House of Representatives by making a defense that you can designate a civilian broadcasting organization and have it report what suits the government”. “It does not create any new added value like the manufacturing industry. What is the growth industry in this?” “I think it lacks the dignity of a nation”. Note that this figure is an illustration, but live-action video was used in the experiment.

This material was selected as appropriate for this experiment. The person’s emotional impression in the video was pre-confirmed as approximately 30 points when judged by people on a scale of 100 (0: very negative, 50: neutral, 100: very positive). This was determined because 44 subjects viewed the video under the condition without emotion estimation information, and their average score of the person’s emotions was 3.2 points on a 10-point scale (1: very negative, 5: neutral, 10: very positive). This material was selected as appropriate for this experiment since the material left room for the impression to be manipulated in a more positive (or negative) direction.

### 4.2. Condition for Biases of Emotion Estimation Information

Based on the system in the previous section, we implemented a system for estimating the degree of negativity of an emotion. In this experiment, the index of anger was used as an indicator. The following two conditions were prepared for the emotion estimation information. The presentation screen for each condition is shown in Figure 5B,C. The experimental procedure of setting up two information presentation conditions, High (higher value) and Low (lower value), is based on prior research on the anchoring effect [61,62,63]. In the example of the previous study, the subjects were given a condition in which they were presented with either a higher or lower price when thinking about the price of a car.

- Negative estimation condition. In this condition, a higher degree of negativity of estimation results was presented by using the Negative Estimation AI. Specifically, the estimation results were presented between 0 and 10 points (i.e., very negative). These estimation results were more negative than most subjects’ self-judgments since the pre-confirmed emotional negativity degree in the condition without the emotion estimation information was about 30 points as mentioned earlier. Since the raw values of the Anger index were calculated on a scale from 0 to 100, the values were re-scaled from 90 to 100 points. The raw values of the anger index were also adjusted by applying a multiplication to the raw values so that the average value of the estimation results for the entire video would be 5 points.
- Positive estimation condition. In this condition, a lesser degree of negativity (i.e., a more positive degree) of estimation results was presented by using the positive estimation AI. Specifically, the estimation results were presented between 40 and 60 points (i.e., neutral). These estimation results were more positive than most subjects’ self-judgments. As the same manner as the negative estimation condition, the raw values of the anger index were re-scaled from 40 to 60 points. The raw values were also adjusted so that the average value of the estimation results for the entire video would be 50 points.

### 4.3. Procedure

**Figure 6:**
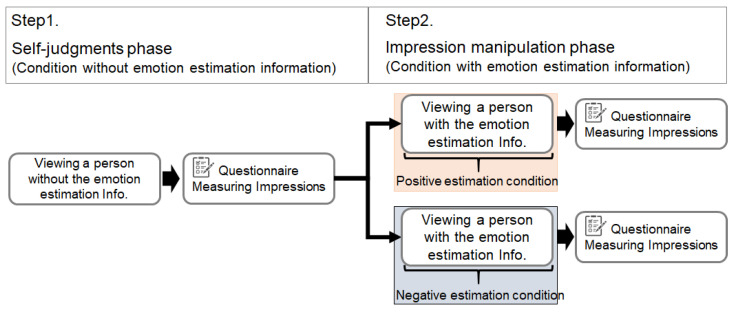
Protocol of the experiment.

The experimental protocol is shown in Figure 6. The procedure consists of two parts.

Step 1. The self-judgments phase (i.e., condition without emotion estimation information). First, the subjects viewed the video without emotion estimation information. Then, the subjects answered a questionnaire about their impressions of the politicians. This answer becomes the subject’s self-judgment. There were three questionnaire items on a 10-point (1: very negative, 5: neutral, 10: very positive) scale, which were impressions (i.e., how they feel and see) of a person regarding emotions, facial expressions, and words and actions. The subjects were then divided into two groups for Step 2 so that there was no bias in the questionnaire scores between the groups.

Step 2. The impression manipulation phase (i.e., condition with emotion estimation information). Next, the same videos as in Step 1 were viewed with the emotion estimation information. Different information was presented for each condition. Then, the same questionnaire as in Step 1 was conducted. This answer becomes the decision after viewing the emotion estimation information. Before Step 2, the emotion estimation information was explained as follows: the emotion was estimated on a 100-point scale (1: very negative, 100: very positive), and the estimation was conducted by artificial intelligence based on machine learning.

### 4.4. Result

**Figure 7:**
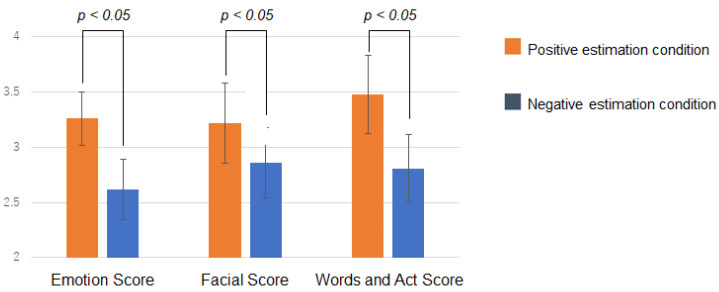
Results in Experiment 1. The questionnaire scores for each bias condition after viewing the information.

An analysis of variance (ANOVA) of the three factors was performed. The three factors were: the bias condition of the emotion estimation information (i.e., positive and negative estimation conditions), the viewing condition of the emotion estimation information (i.e., before and after viewing the emotion estimation information), and the questionnaire items. ANOVA results showed an interaction between the bias condition and viewing condition (F(1,264)=4.19, p< 0.05). The results of the interaction analysis were as follows. There was a significant difference in questionnaire scores between the bias conditions after viewing the emotion estimation information (F(1,264)=6.32, p< 0.05). Figure 7 shows the questionnaire scores for each bias condition after viewing the information. Error bars indicate standard errors. A smaller value means that the impression was negatively influenced. For the questionnaire scores, the negative estimation condition resulted in lower values and the positive estimation condition resulted in higher values.

**Figure 8:**
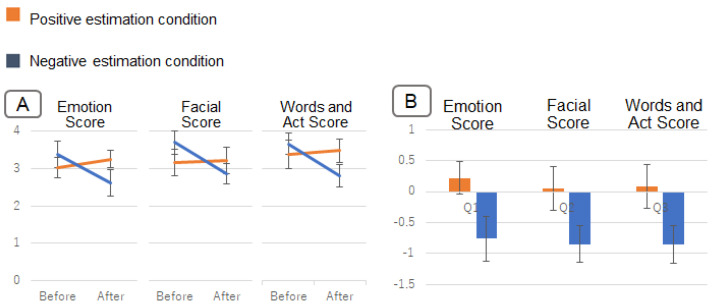
Results in Experiment 1. (**A**) shows the change in questionnaire scores before and after viewing the information. (**B**) shows the amount of change in the questionnaire score from before to after viewing the information.

Next, Figure 8A shows the change in questionnaire scores before and after viewing the information. Figure 8B shows the amount of change in the questionnaire score from before to after viewing the information. This is the after-viewing score minus the before-viewing score. Error bars indicate standard errors. There was no significant difference between before-viewing and after-viewing, but the questionnaire scores changed in a direction consistent with the presented information. Questionnaire scores for the negative estimation condition changed more negatively after viewing the information than before viewing it. Although the amount of change was smaller than in the negative estimation condition, questionnaire scores in the positive estimation condition also changed more positively after viewing the information than before viewing it.

### 4.5. Discussion

The results supported the hypothesis of this study. Viewing emotion estimation information affected the impressions (i.e., how they felt and saw) of the person with negative facial expressions and behaviors. Specific trend changes in impressions occurred in response to different estimated information. As questionnaire scores after viewing the information shown in Figure 7 show, the subjects in the negative estimation condition got the impression more negatively, and the subjects in the positive estimation condition got the impression more positively. This result indicated that people’s cognition and psychology changed unconsciously, consistent with the emotion estimation information they viewed.

The results also indicated that the impression of negative facial expressions and speech and behavior could change in either a more negative or less negative (i.e., positive) direction upon viewing the emotion estimation information. The change in questionnaire scores after viewing the information can be interpreted as the overriding of self-judgments by the AI emotion estimation information. However, since there was no significant difference between before and after viewing the information, it can be interpreted that the degree of change was small, and in particular, the impression was not likely to change in the direction of weakening negativity (i.e., positive direction) for the person in this video. This result also indicated that the change in the impression of emotion caused by viewing the emotion estimation information spill over to the impression of behavior and facial expressions. This result can be seen from results in which all scores tended to change with the same trend. For example, in the negative estimation condition, it can be interpreted that the target person’s emotion was felt more negatively, which also made the person’s facial expressions, speech, and behavior appear more negative (e.g., anger).

## 5. Evaluation 2: Verification of Impression Manipulation of a Person with Positive Facial Expressions

Unlike Evaluation 1, Evaluation 2 verified the hypotheses on the person with positive facial expressions. The 36 subjects were Japanese. Subjects were college students in their early 20s and were collected through an open recruitment process at the university. The experimental procedure was the same as in Evaluation 1.

### 5.1. Positive Facial Expression Person

**Figure 9:**
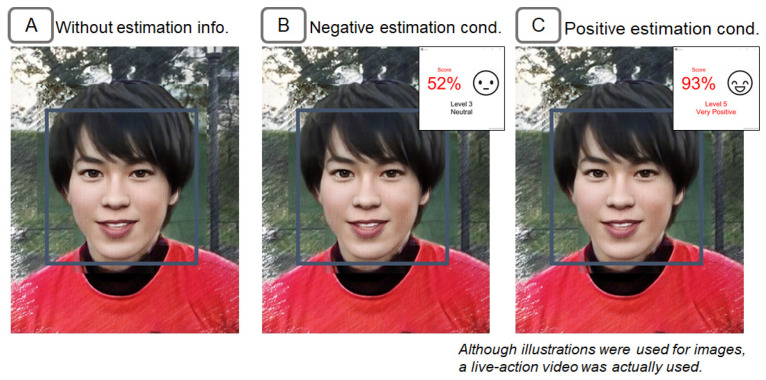
Presentation information for the positive facial expression man in Experiment 2. (**A**) Screen with no emotion estimation information, (**B**) screen with negative estimation information, and (**C**) screen with positive estimation information.

The subject of the impression manipulation was a young man answering interviews about success experiences after running events. The video was a 30 s video selected from running event report videos in Japan. In the video, one young man was shown in the center of the image as shown in Figure 9A. His facial expression was positive. He expresses positive impressions about his words and actions. For example, the words include the followings. “I was able to keep up with Mr. Tanaka, who came in first, all the way to the end, so my self-esteem became high”. “It felt good”. “Tanaka-san, who came in first, was very fast. Well, I managed to keep up with him and came in second. Ha-ha-ha”.

This material was selected as appropriate based on the same criteria as in Evaluation 1. The person’s emotional impression in the video was pre-confirmed as approximately 80 points when judged by people on a scale of 100. This was determined because 36 subjects viewed the video under the condition without emotion estimation information, and their average score of the person’s emotions was 8.4 points on a 10-point scale.

### 5.2. Condition for Biases of Emotion Estimation Information

We implemented a system for estimating the degree of positivity of an emotion. In this experiment, the index of happy was used as an indicator. The following two conditions were prepared for the emotion estimation information. The presentation screen for each condition is shown in Figure 9B,C.

- Negative estimation condition. In this condition, a less degree of positivity (i.e., a more negative degree) of estimation results was presented by using the negative estimation AI. Specifically, the estimation results were presented between 40 and 60 points (i.e., neutral). These estimation results were more negative than most subjects’ self-judgments since the subject’s emotional degree was about 80 points, as mentioned earlier. Since the raw values of the happy index were calculated on a scale from 0 to 100, the values were re-scaled from 40 to 60 points. The raw values of the happy index were also adjusted by applying a multiplication to the raw values so that the average value of the estimation results for the entire video would be 50 points.
- Positive estimation condition. In this condition, a higher degree of positivity of the estimation results was presented by using the positive estimation AI. Specifically, the estimation results were presented between 90 and 100 points (i.e., very positive). These estimation results were more positive than most subjects’ self-judgments. The raw values were re-scaled from 90 to 100 points, similar to the negative estimation condition. The raw values were also adjusted so that the average value of the estimation results for the entire video would be 95 points.

### 5.3. Result

**Figure 10:**
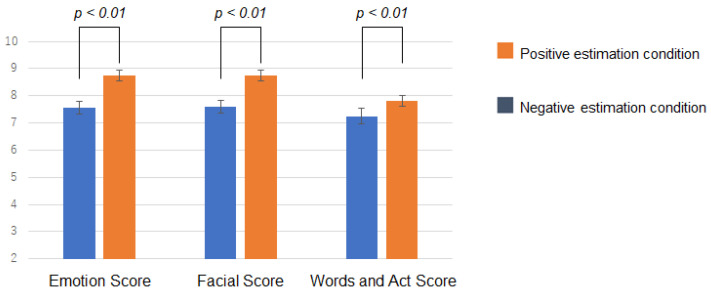
Results in Experiment 2. The questionnaire scores for each bias condition after viewing the information.

ANOVA of the three factors was performed as the same as Evaluation 1. ANOVA results showed an interaction between the bias condition and viewing condition (F(1,215)=6.76, p< 0.05). The results of the interaction analysis were as follows. There was a significant difference in questionnaire scores between the bias conditions after viewing the emotion estimation information (F(1,215)=25.70, *p* < 0.01). Figure 10 shows the questionnaire scores for each bias condition after viewing the information. A larger value means that the impression was positively influenced. For the questionnaire scores, the negative estimation condition resulted in lower values and the positive estimation condition resulted in higher values.

**Figure 11:**
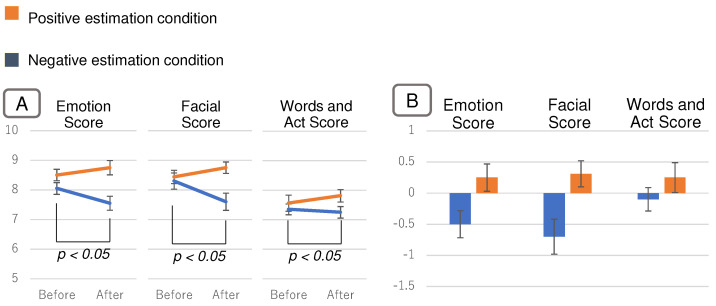
Results in Experiment 2. (**A**) shows the change in questionnaire scores before and after viewing the information. (**B**) shows the amount of change in the questionnaire score from before to after viewing the information.

Next, there was a significant difference in questionnaire scores between before and after viewing in the negative estimation condition (F(1,215)=5.12, p<0.05). Figure 11A shows the change in questionnaire scores before and after viewing the information. Figure 11B shows the amount of change in the questionnaire score from before to after viewing the information. This is the after-viewing score minus the before-viewing score. Questionnaire scores for the negative estimation condition changed more negatively (i.e., less positively) after viewing the information than before viewing it. Although there was no significant difference, questionnaire scores in the positive estimation condition also changed more positively after viewing the information than before viewing it.

### 5.4. Discussion

Phenomena that support the hypothesis of this study also occurred for the person with positive facial expressions and behaviors. As shown in Figure 10, there was a significant difference in questionnaire scores after viewing the information between the negative and positive estimation conditions. This indicates that viewing the emotion estimation information changes the impression of the person with positive facial expressions. From the negative estimation condition in Figure 11, it can be inferred that the impression of the person in this experiment was easier to change in the negative direction (in the direction of reduced positivity). In addition, these results also indicate that the effect caused by the emotion estimation AI spills over to not only the impression of emotion, but also the impression of facial expressions, words, and behavior.

## 6. Evaluation 3: Verification of Impression Manipulation of a Person with Neutral Facial Expressions

In Evaluations 1 and 2, we could not identify any examples of significant changes in impressions toward the positive direction from before to after viewing the emotion estimation information. Therefore, Evaluation 3 verified whether the impression of a person with neutral facial expressions could be manipulated in a positive direction in a scenario that aims to change the employment interviewer’s impression to a more positive one. The 17 subjects were Japanese. The subjects were college students in their early 20s and were collected through an open recruitment process at the university. Although the experimental procedure was the same as in Evaluations 1 and 2, the bias condition for the emotion estimation information was only the positive estimation condition of Evaluation 2.

### 6.1. Neutral Facial Expression Person

**Figure 12:**
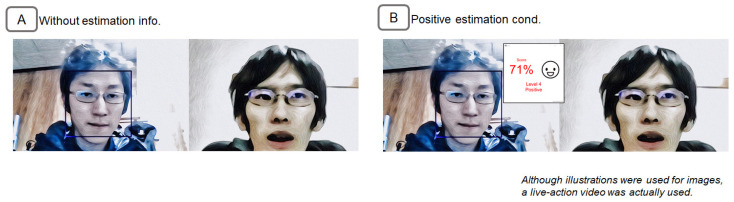
Presentation information for the neutral facial expression interviewer in Experiment 3. (**A**) Screen with no emotion estimation information. (**B**) Screen with positive estimation information.

The subject of the impression manipulation was an interviewer who asked questions to job applicants in employment interview situations. The video was a 30 s video created using two extras. In the video, the interviewer was on the left side of the screen and the job applicant was on the right side of the screen, as shown in Figure 12A. The interviewer’s facial expression was neutral. The interviewer asks the job applicant about his motivation for applying for the job and listens to the job applicant’s response. The content of the conversation includes the following. Interviewer: “Now, the first person. Please answer why you are interested in our company”. Applicant “Yes, I am interested in AR technology and wanted to research and develop using AR technology, so I applied to your company”. Interviewer “Okay, please tell me about the technology you use daily”. Aspirant: “Yes, I develop applications using AR technology, mainly in the iOS environment”.

This material was selected as appropriate based on the same criteria as in Evaluation 1. The person’s emotional impression in the video was pre-confirmed as approximately 45 points when judged by people on a scale of 100 points. This was determined because 17 subjects viewed the video under the condition without emotion estimation information, and their average score of the person’s emotions was 4.6 points on a 10-point scale.

### 6.2. Result

**Figure 13:**
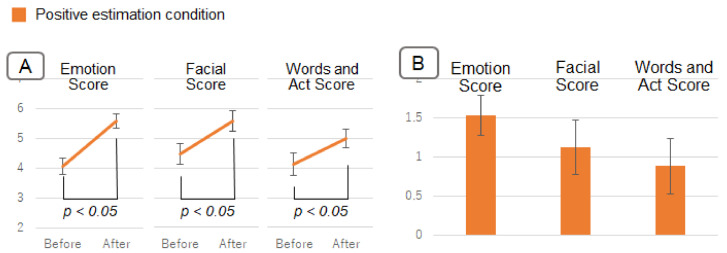
Results in Experiment 3. (**A**) shows the change in questionnaire scores before and after viewing the information. (**B**) shows the amount of change in the questionnaire score from before to after viewing the information.

ANOVA of the two factors was performed. The two factors were: the viewing condition of the emotion estimation information and the questionnaire items. ANOVA results showed a significant difference between before and after viewing the emotion estimation information (F(1,101)=27.56, p<0.01). Figure 13A shows the change in questionnaire scores before and after viewing the information. Figure 13B shows the amount of change in the questionnaire score from before to after viewing the information. There was a main effect for the viewing condition (F(1,101)=27.56, p<0.01). In the positive estimation condition, questioner scores after viewing the information were more positive than those before viewing it. There was also a main effect for the questionnaire items (F(2,101)=5.63, p<0.01). The results of the multiple comparison test using the Bonferroni method showed that the emotion score was significantly higher than the words and actions score (p<0.05).

### 6.3. Discussion

In the scenario of changing the interviewer’s impression to a more positive one, the neutral facial expression interviewer’s impression was significantly changed in a positive direction by the positive estimation condition. This result indicates the feasibility of user assistance using the unconscious positive impression change about the interlocutor. For example, suppose a communication situation such as an interview where users should not increase their psychological burden. In this case, a negative impression of the interlocutor may make the user feel more intimidated by the interlocutor’s words, behavior, and emotion, which may increase the user’s psychological burden (e.g., anxiety level and tension level) and cause a decrease in dialogue performance (e.g., response failure). For such situations, it can be assumed that using the proposed method to positively change the interlocutor’s impression may lead to a decrease in psychological burden and an improvement in dialogue performance.

## 7. General Discussion

The outcome of this research is to demonstrate a phenomenon and manipulation method of unconscious cognitive and psychological change caused by AI’s emotion estimated information containing a bias.

Case 1 in Section 3 (i.e., the section of a method) was rejected. Results showed that viewing AI-estimated information affects people. Case 2 was then rejected, and Case 3 was accepted. Phenomena could occur in a predictable trend depending on the different conditions of the emotion estimation information. The phenomena could be manipulated intentionally. Therefore, it was found necessary to consider the impact of this phenomenon.

The results indicate that people have cognitive biases in which their judgments of others’ impressions are influenced by viewing AI emotion estimation information. It can be seen from the result that subjects’ cognition and psychology changed unconsciously to match the emotion estimation information they viewed, and it can also be seen from the results that self-judgments of others’ impressions were overridden by viewing AI-estimated information. Thus, viewing the information of estimating positively (or negatively) other people’s emotions leads to the phenomenon of perceiving other people’s impressions positively (or negatively). It can be assumed that this phenomenon does not occur due to individual differences and different information viewing scenarios.

Possibility of manipulation of the phenomenon: Results showed that the cognitive bias caused by AI emotion estimation information could occur with a predictable tendency. This can be seen from the results that the subject’s impression could be manipulated in either a more negative or more positive direction, depending on the two types of information presentation conditions provided in this experiment. This means that biases occurred in the subject’s cognition with the same tendency as the biases within the emotion estimation AI. These results strongly indicate the possibility of intentionally manipulating this phenomenon to change people’s impressions, either accidentally or intentionally.

The possibility that the change in an impression of emotion caused by this phenomenon spills over to other impression changes: The impression of others’ emotions was changed by viewing the emotion estimation information, and the same tendency was observed in how to feel other aspects (e.g., facial expressions, words and actions). In other words, when the other’s emotions were seen more positively or negatively, the other’s facial expressions and words and actions were also seen as positively or negatively. There is a possibility that such changes in the impressions may affect a people’s psychological and psychosomatic state. For example, gaining a negative impression of the other person may change the way people respond to the other person or may gain a psychological burden during conversations with the other person. These results indicate that the influence of the emotion-estimation AI may spill over to impressions of not only emotions, but also other aspects.

Ripple effects: The phenomena we focused on are assumed to occur in situations where emotion estimation AI is used. In the future, people who find it more useful to know the emotional status of others will install emotion estimation systems in wearable and mobile information devices so that they can automatically view the estimated information of others in most situations where they see others’ faces. As AI will spread in many aspects of daily life, the phenomena confirmed in this study can be assumed to cause many small changes and have a large impact on people without noticing it.

We consider that the unconscious effects caused by viewing the emotion estimation information can occur automatically and unconsciously. Such effects are assumed to be caused by an automatic mind [64,65] that is defined in the dual-process theory which classifies the mind and brain into two systems. The automatic mind often leads to irrational and subjective responses, which are interpreted in terms of cognitive biases, illusions, and psychological phenomena. In contrast, a reflected mind [65,66] is controlled by the person’s consciousness and is difficult to prevent the unconscious phenomena caused by the automatic mind. Therefore, the unconscious effects caused by viewing the emotion estimation information are assumed to be difficult to prevent. The phenomenon in which cognition and psychology change to match the emotion estimation information is considered to be a phenomenon similar to the anchoring [62,63,67] or placebo effect [68].

### 7.1. Dark Side: Possible Adverse Effects of Cognitive Biases Caused by Browsing AI Estimated Information

This paper shows that the presentation of emotion estimation information can change the way people feel and see other people unconsciously. This indicates that the presentation system of AI emotion estimation information may have a negative impact on people, i.e., it may change people’s cognition and psychology in a direction that causes problems. Examples of possible problems include the following. Impressions about people (e.g., politicians, celebrities, people close to them) are changed, which leads to public sentiment induction and propaganda. How communication partners (e.g., friends, business partners) feel and see each other can change, which leads to a decrease in the quality of cooperative work, interpersonal conflicts, disagreements, etc. In an employment interview situation, the interviewer’s words and actions are seen as negative, which increases applicants’ psychological stress and leads to the failure of the interview. One’s own emotions are perceived as negative when looking at oneself, which actually changes one’s mental state and behavior negatively. People are unaware of their interlocutor’s negative emotions, which leads to making the situation worse. Although Ms. Risa performs a neutral treatment toward Mr. Jane of the opposite sex, whom Ms. Risa does not like, Mr. Jane perceives Ms. Risa’s behavior positively and causes problems for Ms. Risa. In the future, one of the factors that cause the fragmentation of any point (e.g., mental health or thinking) in a group (e.g., country) may be concluded that different emotion estimation AI is used by default in each group. Since various factors (e.g., training data set, how the data is labeled, developer adjustments) can change the AI’s emotion estimation bias, the occurrence of different cognitive biases in different groups using different emotion estimation AIs can be a factor in the problems mentioned above.

It can be assumed that these problems can occur either accidentally (unexpectedly) or intentionally by someone’s malicious intent to benefit a particular country, organization, or individual (e.g., a mechanism is installed that causes a bad phenomenon).

These problems need to be considered when using and designing emotion estimation systems in the future. The following is an example to reduce the problem. The following is an example: A simple test to understand the changes in himself/herself is developed, and the test is conducted before using the system at regular intervals (e.g., in units of several months). For each system use situation, the system works a mechanism that does not allow phenomena to occur in the direction where problems occur. For example, in situations where it is prohibited to cause a negative perception or appearance of each other, a mechanism that makes it difficult for such phenomena to occur (e.g., a mechanism that makes it possible to easily perceive each other positively) should be installed.

The results of this research are helpful for predicting one of the negative effects caused by emotion estimation AI. In addition, the proposed method (i.e., a method for inflicting bias on emotion-estimation AI) is helpful in predicting how impression manipulation using emotion-estimation AI can be caused by malicious organizations or individuals. These findings are helpful for users and designers of emotion estimation AI and other AI systems that handle human estimation information.

### 7.2. Light Side: Feasibility of Technology That Supports Users by Utilizing Cognitive Biases Caused by Browsing AI Estimated Information

There is no right or wrong in which the emotional estimation information of AI causes cognitive bias in people. A helpful idea would be to accept this phenomenon and use this cognitive bias for a good purpose.

This paper demonstrates the possibility of utilizing this phenomenon positively. The results indicated that the proposed method may be helpful as an easy way to unconsciously change how people see and feel toward other people by taking advantage of the cognitive bias caused by AI emotion estimation information. By nature, it is difficult for people to change how they perceive each other. However, by using the proposed method, such changes can quickly occur. It is also noteworthy that the proposed method caused the phenomenon instantaneously and did not require any additional effort from the user. It can be assumed that how people see and feel about other people can affect their mental state, their behavior, and the performance they can exhibit. Therefore, our proposed method is considered to be used for good purposes in situations viewing people. For example, a previous study has shown that the quality of communication and creative collaborative work results with others is improved in environments where the facial expressions of communication partners appear positively by using VR technology [26]. A similar phenomenon as this previous study is assumed to be controlled by using the proposed method. In addition, in situations such as important discussions or interviews, making the other person appear positively through viewing emotion-estimated information is assumed to reduce psychological burden (e.g., tension, anxiety) and support people. In fact, the previous study has been conducted to eliminate negative impressions (e.g., fear) of the opponent during interviews and debates [9]. Moreover, there are both problems caused by the inability to convey his/her intended emotions to the other person and problems caused by the effort and fatigue required to convey his/her intended emotions to the other person (e.g., emotional labor). To address these problems, it is assumed to be an effective support to make his/her intended emotion visible to the other person with AI emotion estimation information during communication.

The technology demonstrated in this study shows the possibility to use the cognitive bias caused by emotion estimation AI for positive purposes.

### 7.3. Future Work

(1) To generalize the findings of this study to a broader population, we plan to evaluate a more diverse demographic of subjects since the subjects of our paper were young Asians. (2) We also plan to study mechanisms to prevent the phenomenon we have focused on from having a negative impact on people. (3) In addition, we plan to develop and verify applications that utilize the proposed method for positive purposes. (4) Furthermore, we plan to investigate which individual characteristics are associated with susceptibility to this phenomenon and develop a mechanism to detect and deal with susceptible people in advance. (5) In this study, we investigated cognitive bias when the subject perceives the source of information for the emotion estimation score as AI. On the other hand, technology that replaces this information source as something other than AI (e.g., other people’s evaluations) may spread. Therefore, it is also necessary for the future to investigate how cognitive bias occurs depending on the user’s perception of the information source. (6) Although the emotion estimation technology in this experiment used only visual information (camera images), there are emotion estimation technologies that use sensor data from multiple modalities, such as vision and voice. In the future, users may view the person with technologies that directly modify the person’s appearance (facial expressions) and voice. It is necessary to investigate how these various technologies cause cognitive bias regarding emotion estimation in the future.

## 8. Conclusions

This study examined the existence and manipulation method of cognitive bias caused by viewing biased AI’s emotion estimation information. The emotion estimation system using the proposed method had a bias that made the emotion estimation more positive/negative than usual. A prototype system was implemented and our hypothesis was evaluated for impressions of persons with negative facial expressions, positive facial expressions, and neutral facial expressions in the videos. The results showed that viewing information that estimated others’ emotions more positively/negatively caused the phenomenon in which the user’s self-judgment was overridden and others’ impressions of emotions, words, and actions were perceived more positively/negatively. These results support our hypothesis and indicate that biased emotion estimation information causes a phenomenon that quickly and unconsciously changes the way people see and feel others’ impressions. The existence of these phenomena and methods indicates that a biased emotion estimation AI may accidentally or intentionally cause adverse effects on people and society through the manipulation of impressions. In addition, their existence indicates the possibility that a biased emotion estimation AI can be used for good purposes to support users. This study helps predict what a biased emotion estimation AI might affect people and provides helpful insights for the design and the use of emotion estimation AI considering cognitive biases.
